# Novel Action of FSH on Stem Cells in Adult Mammalian Ovary Induces Postnatal Oogenesis and Primordial Follicle Assembly

**DOI:** 10.1155/2016/5096596

**Published:** 2015-11-09

**Authors:** Deepa Bhartiya, Seema Parte, Hiren Patel, Kalpana Sriraman, Kusum Zaveri, Indira Hinduja

**Affiliations:** ^1^Stem Cell Biology Department, National Institute for Research in Reproductive Health, JM Street, Parel, Mumbai 400 012, India; ^2^PD Hinduja Hospital, Veer Savarkar Marg, Mahim, Mumbai 400016, India

## Abstract

Adult mammalian ovary has been under the scanner for more than a decade now since it was proposed to harbor stem cells that undergo postnatal oogenesis during reproductive period like spermatogenesis in testis. Stem cells are located in the ovary surface epithelium and exist in adult and menopausal ovary as well as in ovary with premature failure. Stem cells comprise two distinct populations including spherical, very small embryonic-like stem cells (VSELs which express nuclear OCT-4 and other pluripotent and primordial germ cells specific markers) and slightly bigger ovarian germ stem cells (OGSCs with cytoplasmic OCT-4 which are equivalent to spermatogonial stem cells in the testes). These stem cells have the ability to spontaneously differentiate into oocyte-like structures *in vitro* and on exposure to a younger healthy niche. Bone marrow may be an alternative source of these stem cells. The stem cells express FSHR and respond to FSH by undergoing self-renewal, clonal expansion, and initiating neo-oogenesis and primordial follicle assembly. VSELs are relatively quiescent and were recently reported to survive chemotherapy and initiate oogenesis in mice when exposed to FSH. This emerging understanding and further research in the field will help evolving novel strategies to manage ovarian pathologies and also towards oncofertility.

## 1. Introduction

The central dogma of reproductive biology that ovary has fixed number of follicles at birth or shortly afterwards was first put forth by Heinrich Waldeyer, a German anatomist-embryologist in 1870. It stated that a woman is born with a finite and nonrenewing pool of germ cells whose numbers decline progressively with age, affecting ovarian function and sudden demise of follicles with age results in menopause. Besides the fixed number of follicles in the ovary, it is also a well-established fact that ovarian function is modulated by pituitary gonadotropins follicle stimulating hormone (FSH) and luteinizing hormone (LH). FSH acts on growing follicles through its receptors (FSHR) located on the granulosa cells and initial follicle growth particularly in women is gonadotropin independent [1]. LH is responsible for ovulation and synthesis of steroid hormones.

The concept of biological clock of ovary and that a female is born with a fixed number of follicles was challenged in 2004 by Professor Tilly and his group who rekindled the very essence of the topic of postnatal oogenesis and presented evidence that the rate of loss of oocytes in mice ovary due to atresia and ovulation were indeed counterbalanced by a mechanism which maintains a constant count of immature oocytes [2]. These observations favored the concept of ovarian stem cells and postnatal oogenesis and several groups were drawn into this area of research.

First major step was to prove the presence of stem cells in the ovary and their characterization, followed by how they function under normal conditions leading to postnatal oogenesis, and how they result in various pathologies like ovarian failure, menopause, and cancer. Also, it became pertinent to study whether stem cells present in the adult ovary could be manipulated to regain ovarian function under certain specific conditions, for example, after oncotherapy in cancer survivors. Postnatal follicular regeneration in mouse ovary [3] and ovary surface epithelium (OSE) as a source of germ cells during fetal stage ovary was reported in the past [4, 5]. It was also proposed that OSE is the active site of origin for neoplasms and almost 90% of ovarian cancers arise from the OSE [6]. Various other methods like label retaining cells, Hoechst dye-excluding side population confirmed the presence of stem/ progenitor cells [7–9] and a novel population of stem-like cells coexpressing Lin28 and Oct-4 in epithelial ovarian cancers have been reported [10]. Flesken-Nikitin et al. [11] showed the presence of stem cells in the OSE in the hilum region as the niche for ovarian cancer cells.

Present review provides a brief overview of our current understanding on ovarian stem cells, their origin and characterization, and how they are implicated in postnatal oogenesis along with an interesting advance from the authors' laboratory that they express follicle stimulating hormone receptors (FSHR) and are modulated by FSH to undergo self-renewal, clonal expansion to form germ cell nests, proliferation, differentiation, and primordial follicle (PF) assembly in adult ovary. It also touches upon subtle technical issues that should be kept in mind to arrive at a consensus on existence of stem cells in adult mammalian ovary.

## 2. Stem Cells, Progenitors, and Germ Cell Nests in Adult Mammalian Ovary

Ovary is a dynamic organ lined by a single layer of cuboidal surface epithelial cells also called germinal epithelium which is relatively less differentiated and uncommitted and express epithelial and mesenchymal markers under normal conditions. OSE is involved in follicular rupture, release of the mature oocyte, subsequent ovarian remodeling, and repair of follicle walls and hence becomes a discontinuous layer in case of anovulatory cycles anovulatory cycles, polycystic ovarian syndrome and during menopause and in sclerotic ovaries [6]. First proof for the presence of ovarian stem cells in OSE was given by Tilly's group [2] when they showed MVH and BrdU coexpressing cells in the OSE along with meiotic markers (Scp3, Spo11, and Dmc1) and that on grafting wild type ovary in GFP mice led to formation of follicles with GFP oocyte enclosed by wild-type granulosa cells. Thereafter various research groups became involved and investigated ovarian stem cells with the help of varying approaches like immunomagnetic antibody and flow cytometry based cell sorting strategies (MACS and FACS),* in vitro* culture and differentiation of ovarian stem cells, genetic lineage tracing, and transplantation experiments, suggesting that the follicle pool is not a static but indeed a dynamic population of differentiating and regressing structure in adult mice and human ovary. Main highlights of various studies that were undertaken to show the presence of stem cells in adult ovaries have been compiled in Table 1 [13–35].

As evident, Tilly's and Bukovsky's groups were the first to report stem cells in adult mammalian OSE. Bukovsky's group reported spontaneous differentiation of oocyte-like structures* in vitro* from OSE cells for the first time and both the groups reported beneficial effect of bone marrow cells on ovarian function. Tilly's and Virant-Klun's groups detected stem cells in menopausal and POF women. The former group could restore ovarian function by providing a young niche in mice while the latter group demonstrated spontaneous differentiation of human OSE cells to oocyte-like structures* in vitro*. Using a handful of characteristic markers (meiotic and germ cells, PGCs, and primordial oocyte specific), Tilly's group has been successful to demonstrate PF assembly in cortical tissue slices* in vitro* [21]. Virant-Klun's group has extensively characterized the ovarian stem cells and reported the presence of spherical, very small 4 *μ*m cells which express pluripotent and PGC specific markers [36]. Johnson et al. [18] also detected PGCs (Stella, Fragilis, and Nobox) and germ cells (Oct4, Mvh, and Dazl) specific transcripts in bone marrow.

**Figure 1:**
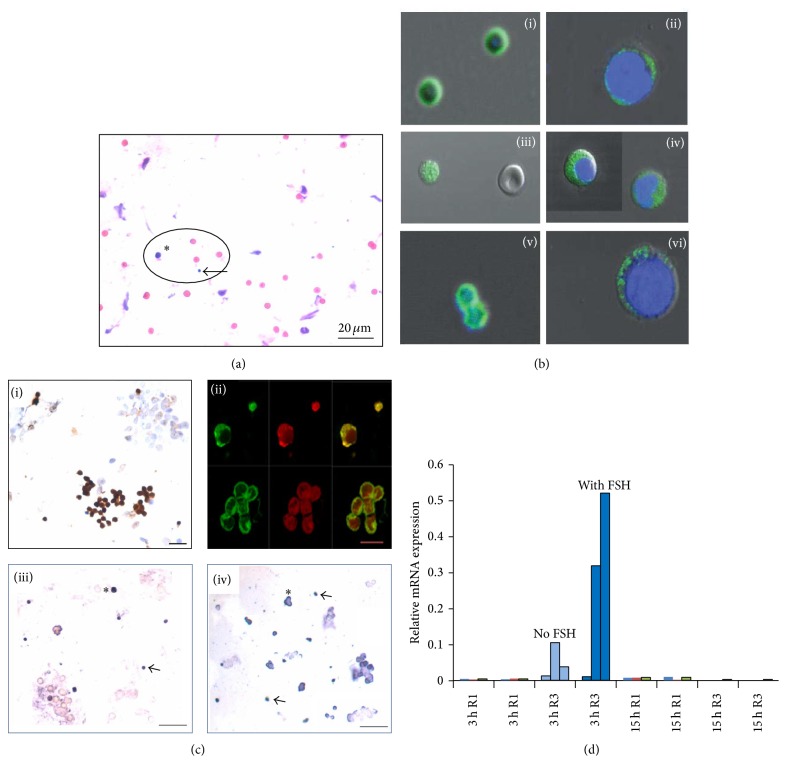
FSH-FSHR3-stem cell interaction in ovary surface epithelium. (a) H&E stained sheep OSE smear. Two distinct populations of stem cells (encircled) including VSELs (arrow) which are smaller than the red blood cells and slightly bigger OGSCs (asterix) are clearly visualized even after gently scraping sheep ovary fixed overnight in neutral buffered formalin. Red blood cells and epithelial cells are also clearly visualized [12]. (b) (i)–(vi) Characterization of ovarian stem cells using pluripotent OCT-4 and SSEA4 markers. Smaller VSELs express nuclear OCT-4 and cell surface SSEA4 whereas slightly bigger OGSCs express cytoplasmic OCT-4 and minimal SSEA4. Note the VSELs do not stain with DAPI [13]. (c) (i) Sheep OSE smear immunostained with FSHR antibody. Note epithelial cells are negative whereas the stem cells exhibit distinct positive stain. (ii) Confocal microcopy localization of FSHR on VSELs and OGSCs and on a germ cell nest. (iii)-(iv)* In situ* hybridization of FSHR on ovarian stem cells after FSH treatment using oligo probes specific for FSHR1 and FSHR3, respectively. Note active transcription of FSHR3 mRNA in the cytoplasm of stem cells after FSH treatment whereas FSHR1 is expressed in the stem cells and the expression is not affected by FSH treatment. (d) qRT-PCR analysis of FSHR1 and FSHR3 after 3 and 15 h of FSH treatment. Note that only FSHR3 levels are increased transiently after 3 and return to basal levels by 15 h. (c) and (d) Panels show earlier published from 3 different experiments represented individually by Patel et al. [14]. Please refer to the cited references for further details.

Our group initially obtained institutional ethical approval to use human ovarian cortical tissue to establish methods to cryopreserve PF for cancer patients. But few samples analyzed by us revealed nil follicles/germ cells in the ovarian tissue. Then with technical help from Professor Bukovsky, we initially reported that adult rabbit, sheep, monkey, and human OSE harbored stem cells and for the first time demonstrated that there existed two distinct populations of stem cells in OSE including (i) spherical cells which were smaller than RBCs in agreement with Virant-Klun's observations and (ii) a slightly bigger population of “progenitors.” Immunolocalization studies showed that the smaller cells were pluripotent and expressed nuclear OCT-4, whereas the bigger ones expressed cytoplasmic OCT-4 (Figure 1). A careful survey of literature showed that Professor Ratajczak's group had reported similar cells termed very small embryonic-like stem cells (VSELs) in various adult tissues [37]. Thus we realized that the smaller cells with nuclear OCT-4 were the VSELs and when they entered differentiation, nuclear OCT-4 was no longer required, shifted to cytoplasm, and eventually degraded as cells became more committed. The cells with cytoplasmic OCT-4 were termed ovarian germ stem cells (OGSCs) and appear to be similar to the oogonial stem cells (OSCs) reported by Tilly's group. We have reported similar VSELs in adult mammalian testes [38] as well and the OGSCs (OSCs) are equivalent to SSCs in the testes [12, 39, 40].

A careful examination of scraped OSE cells shows the presence of VSELs, OGSCs, and also occasional germ cell nests (GCN) which are formed by rapid, clonal expansion with incomplete cytokinesis of OGSCs. The detailed protocols to study these cells (VSELs, OGSCs, and GCN) by mechanical scraping of bigger sized mammalian ovaries and after enzymatic digestion of mouse OSE were recently described by us [12]. These cell types can also be successfully scraped from a sheep ovary fixed overnight in neutral buffered formalin (Figure 1(a)). Presence of germ cell markers in bone marrow and expression of PGC markers on these stem cells hints to the presence of a common population of VSELs in bone marrow/peripheral blood and ovary as suggested by Ratajczak's group [41].

Presence of stem cells and GCN in adult ovary contradicts the report by Lei and Spradling [42] and technical reasons resulting in the discrepancy have been discussed [34]. Similarly, we also explained as to why Byskov et al. [43] failed to detect stem cells and oogonia in adult human ovary [44]. Zhang and coworkers have published two papers [45, 46] wherein they contradict the presence of stem cells in adult ovary by using elegant lineage tracing studies. We had earlier discussed [34] technical issues related to use of MVH for lineage tracing studies by Zhang et al. [45]. Zhang et al. [46] did not acknowledge our comments, rather discussed the study by Lei and Spradling [42], and have performed further lineage tracing studies to generate evidence against presence of stem cells in adult ovary. But technology can never overtake biology and we have further discussed the study by Zhang et al. [46] in context of our work [16] below.

Yuan et al. [47] have also extensively studied stem cells in monkey ovary by different approaches like flow cytometry, immunolocalization, Western blot, and RT-PCR with completely negative results using testes and fetal ovary tissues as positive control. One thing that needs to be kept in mind is that the rate of turnover of stem cells in testis and fetal ovary is extremely high compared to production of 1-2 oocytes per cycle in adult ovary in women and monkeys and that absence of evidence is not evidence for absence. It is mentioned in the protocols followed in the study that ovarian filtrate was spun at 1200 rpm for further use. We know by our experience that ovarian VSELs and OGSCs do not pellet down when spun at a speed of 1200 rpm; rather we always isolate them by spinning at a speed of 1000 g. Using this speed we have successfully detected 3–5 *μ*m sized VSELs in adult mouse and sheep ovary as LIN−/CD45−/SCA1+ in mice and as OCT4+ in sheep. We could detect 0.02 + 0.008% in normal mouse ovary as LIN−/CD45−/SCA1+ [16]. Similarly 1.26 ± 0.19% of (2–4 *μ*m) and 6.86 ± 0.5% of (4–9 *μ*m) cells expressing OCT-4 were detected in sheep OSE cell suspension [12]. Thus we need to sharpen the technology rather than doubt the biology. Moreover, if the study [47] used as mentioned, one out of every 20 sections (each 5 *μ*m thick), it is very likely ovarian VSELs being 3–5 *μ*m will inadvertently get missed. We still prefer to study ovarian stem cells in carefully scraped OSE cell smears rather than on sections and for flow cytometry/RNA extraction studies care is taken to spin the cells at 1000 g (rather than the standard 1200 rpm speed). Also extracting RNA from whole ovary, to detect few stem cells in OSE, may not be a good approach; rather RNA should be extracted from cortical tissue pieces or scraped OSE cells (various strategies to enrich stem cells).

## 3. Characterization of Ovarian Stem Cells

VSELs are considered to be the descendants of embryonic epiblast derived pluripotent primordial germ cells (PGCs) that, while migrating along the dorsal mesentery to the genital ridge, also gets deposited in various somatic tissues [41]. The VSELs detected in both adult ovaries and testes [36, 40, 48, 49] are probably the primordial germ cells that survive in adult gonads in few numbers. Similar concept that PGCs may survive in adult ovary was put forth by de Felici's group [50] and recently they supported Ratajczak's views that possibly there exists a mixing up of PGCs and hematopoietic precursors during early embryonic development [51]. Thus it was not at all surprising that both Virant-Klun and our group have reported that the VSELs in the adult ovary express both pluripotent and PGC specific markers (Table 1). Similar expression of markers was reported in pig ovary stem cells [52].

VSELs are distinctly spherical, with high nucleocytoplasmic ratio and nuclei stained dark with Hematoxylin and also do not stain easily with DAPI. DAPI is understood to preferentially stain heterochromatin [53, 54] and being pluripotent these cells are expected to have abundant euchromatin. Dark Hematoxylin stained nuclei is characteristic feature of a group of primitive spermatogonia in testis A_dark_ and we observe similar characteristic staining in primitive stem cells in ovary as well. Immunophenotyping studies on sheep OSE revealed the presence of two distinct populations of stem cells including 1.26 ± 0.19% of (2–4 *μ*m) and 6.86 ± 0.5% of (4–9 *μ*m) cells expressing OCT-4 [12]. Similarly, flow cytometry analysis shows that 0.02% + 0.01% cells are LIN−/CD45−/SCA1+ VSELs in normal mouse ovary [16].

However, presence of stem cells in adult mammalian ovary has not yet been well accepted; rather there are groups who have generated evidence against the presence of stem cells in adult ovary and have been also discussed above. This clearly shows that more research is required in the area. Above review of literature and Table 1 show that stem cells do exist in the OSE and it now becomes pertinent to understand how these stem cells function and contribute to postnatal oogenesis in normal adult ovaries. In the next section, various studies done in the authors' lab on how the ovarian stem cells are modulated by FSH are described.

## 4. Novel Action of FSH on Ovarian Stem Cells

The existing dogma in reproductive biology advocates that FSHR in the ovary are expressed exclusively on the granulosa cells and initial follicle growth is gonadotropin independent. Sairam's group has made seminal contributions and shown that sheep ovarian and testicular FSHR undergo alternative splicing resulting in 4 distinct isoforms of which FSHR1 and FSHR3 have biological functions [55]. Babu et al. [56] have reported FSHR isoforms in mouse ovary and their altered expression after PMSG treatment. Both FSHR1 and FSHR3 were detected by RT-PCR in normal ovary and FSHR3 expression was selectively increased after 24 and 48 h of PMSG treatment. Using a FSHR3 specific peptide IgG, Western blotting confirmed the presence and upregulation of FSHR3 in ovary after PMSG treatment. Sullivan et al. [57] have studied relative mRNA expression for alternately spliced FSHR transcripts (FSHR1, FSHR2, and FSHR3) and LHR in small, medium, and preovulatory sheep follicles and have found that FSHR3 is the predominant transcript by qRT-PCR studies and is expressed in higher levels compared to the canonical FSHR1 and that LHR are maximally expressed in the preovulatory follicles. We have found that ovarian stem cells are located in the ovary surface epithelium and express FSHR and that it is the alternatively spliced FSHR isoform FSHR3 which is actively transcribed on being stimulated by FSH and as a result the ovarian stem cells undergo proliferation and clonal expansion to form germ cell nests (Figure 1) [14].

**Figure 2:**
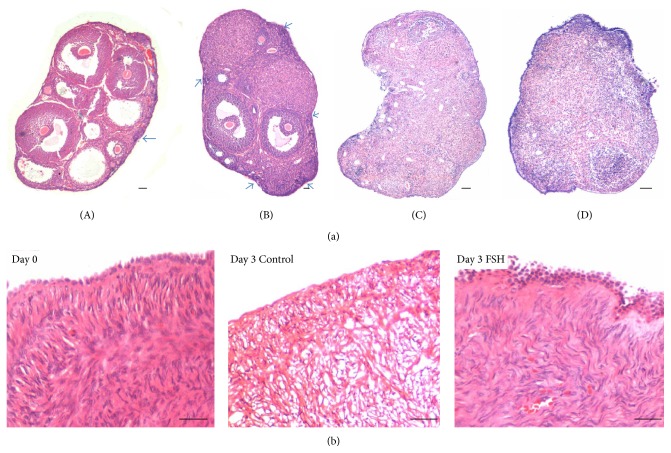
Novel action of FSH on adult mammalian ovary. (a) Effect of pregnant mare serum gonadotropin (PMSG) on intact and chemoablated mice ovaries. (A)-(B) PMSG treatment to intact ovaries results in increased cohorts of primordial follicles below the OSE compared to untreated control [15]. (C)-(D) Chemoablated mouse OSE also responds to PMSG treatment. Note overall thickening of OSE after PMSG treatment compared to untreated control. Chemoablated ovaries are otherwise devoid of follicles [16]. (b) Effect of FSH on human ovary surface epithelium cortical tissue* in vitro* [17]. H&E stained paraffin section on D0 at the start of culture exhibits a prominent OSE and the cortical tissue is almost degenerated by D3 in culture whereas FSH exerts a survival effect on the cortical tissue and note the hypertrophied nature of the OSE. Please refer to the cited references for further details.

These results have relevance especially in light of the fact that no significant association has been observed between mutations or single nucleotide polymorphisms (SNPs) in the canonical FSHR1 with premature ovarian failure and infertility. We have discussed the possible role of FSH-FSHR3-stem cells interaction in the OSE resulting in ovarian cancers, POF, and menopause and how the reproductive biologists have been misled by screening for mutations in FSHR1 with a focus on exon 10 whereas FSHR3 may have a more significant role (has exon 11 and lacks exons 9 & 10) thus explaining the accumulated negative data on lack of mutations in FSHR in women with POF and cancer [58]. Various studies undertaken by us to decipher a novel role of FSH via FSHR3 in stimulating ovarian stem cells (in addition to its well-studied effect on follicular growth via FSHR located on the granulosa cells) resulting in postnatal oogenesis and follicle are listed below:

1. In 2012, we reported [15] that PMSG (FSH analog) treatment to mice stimulates OSE and newer assembly of follicles was evident just below the OSE. PMSG treatment resulted in increased number of PF cohorts compared to normal ovary (Figures 2(A) and 2(B)). A subtle proliferation in OSE was also noted during the proestrus stage of estrus cycle in untreated normal ovary. PMSG treatment augmented this effect and it is crucial to mention here that PMSG shows this effect only when injected during carefully monitored proestrus stage of the estrus cycle.
2. While studying effect of FSH and bFGF on human and marmoset ovarian cortical tissue culture, Parte et al. [17] reported a prominent effect of FSH on OSE (Figure 2(b), became hypertrophied and multilayered) in perimenopausal ovarian tissue and also showed that a large number of stem cells get shed onto the cell culture insert and retained the ability to spontaneously differentiate into oocyte-like structures. qRT-PCR on cortical tissue exposed to FSH and bFGF compared to untreated control showed increased expression of pluripotent (Oct-4A and Nanog), early germ cell (Oct-4, c-Kit, and Vasa), and PF growth initiation (oocyte specific Gdf-9, Lhx8, and granulosa cells specific Amh) markers. Results suggest that FSH exerts a direct action on the stem cells.
3. Patel et al. [14] showed by immunolocalization studies presence of FSHR on the sheep ovarian stem cells whereas the epithelial cells were negative (Figure 1). Using specific oligo probes for* in situ* hybridization studies, they showed that both FSHR1 and FSHR3 mRNA were expressed in the stem cells but only FSHR3 mRNA was actively transcribed and expressed in the cytoplasm of stem cells after FSH treatment. These results were also confirmed by qRT-PCR studies for FSHR1 and FSHR3 transcripts. A prominent effect of FSH was observed on proliferation of stem cells and germ cell nest formation accompanied with upregulation of pluripotent markers Oct4A, Oct4, and Sox2. Thus a functional interaction of FSH-FSHR3-stem cells axis was deciphered for the first time in adult ovary.
4. Ovarian stem cells (VSELs) survive chemotherapy in mice due to their quiescent nature and have the ability to initiate neo-oogenesis on stimulation with FSH. Flow cytometry data shows that LIN−/CD45−/SCA1+ VSELs in normal mouse ovary are 0.02% + 0.01% and after chemoablation are 0.03% + 0.017%. PMSG treatment to chemoablated ovary increased the numbers of VSELs to 0.08 + 0.03% [16].
5. Also a prominent effect of FSH was observed on the OSE of chemoablated ovary after 7 days in culture (Figures 2(C) and 2(D)). BrdU positive cells in OSE were associated with the formation of germ cell clusters. Culture of OSE cells isolated from chemoablated ovary in the presence of FSH resulted in formation of germ cell nests that stained positive for PCNA and OCT-4 and oocyte-like structures* in vitro* that stained positive for MVH and GDF-9 [16].

These data generated by our group suggest a novel action of FSH on the stem cells located in the OSE and call for paradigm shift in the field of reproductive biology. A recent study describes presence of gonadotropin receptors on human bone marrow hematopoietic progenitors including VSELs [59] supporting a developmental link between hematopoiesis and the germline. It becomes extremely perplexing as to how does FSH act on the ovary when an infertility expert treats a woman in the clinic to collect eggs for assisted reproduction. Is FSH really only playing a survival role on ovarian follicles, preventing cell death of a cohort of eggs when they start growing or is it that FSH treatment exerts direct action on the ovarian stem cells and an altogether new cohort of follicles assembles and starts growing starting from the stem cells! We need better means to decipher these well kept secrets of Mother Nature on the surface of the ovary.

Initiation of maturation of the follicles from the primordial pool is a process that at least in humans is not dependent on gonadotropins. Although FSH is a primary factor controlling folliculogenesis, the “initial recruitment” of human PF is mainly controlled by factors produced in the ovaries [1]. In general FSH is secreted in high levels at mid cycle (preovulatory surge) but there is another smaller peak which occurs during late luteal phase and is termed the “intercycle peak” in humans or the “proestrus peak” (secondary surge) in rodents and is understood to be associated with recruitment of follicles for the next cycle. Rani and Moudgal [60] showed that rather than the “preovulatory” FSH peak, the “proestrus” peak affects follicular growth and blocks ovulation in the next cycle. It is probably this intercycle peak of FSH that triggers stem cell activity in the OSE, resulting in PF assembly [15, Figures 1-2] and these follicles then rapidly grow and mature. But more carefully planned studies need to be undertaken to generate more evidence to support this preliminary observation.

Lei and Spradling [42] failed to detect “germline cysts” by lineage tracing approach and thus concluded that adult mouse ovaries lack stem cells. They proposed that primordial follicle pool generated during fetal life is sufficient to sustain oogenesis and that there is no renewal of oocytes during adult life. We had discussed various reasons that could have led to their negative data [34] and also showed presence of “cyst” (germ cell nest) which expresses OCT-4 and SSEA4 in adult mouse OSE cell smears after PMSG treatment. Similar structures have also been characterized in human and sheep ovary [35]. Recently we published our protocols to isolate two populations of ovarian stem cells [12] and we found that it is the bigger OGSCs (equivalent to OSCs) that divide rapidly and form cysts with incomplete cytoplasmic division. Presence of cysts (germ cell nests) is considered to be a hallmark feature of stem cell activity in adult ovary [42].

Recently Zhang et al. [46] have provided compelling evidence against stem cell activity and postnatal oogenesis in adult mice using 3 genetically modified mouse models. Their experiments suggest that there is no generation of oocytes from stem cells in adult ovary and also no somatic cells get recruited to aid primordial follicle assembly with* de novo*-regenerated oocytes. In contrast to their study where genetic manipulations are done to answer a biological question, we used a more physiological approach to address the same question of postnatal oogenesis [16]. Firstly we characterized the stem cells in enzymatically separated OSE cells and used flow cytometry to study LIN−/CD45−/SCA+ VSELs in normal (0.02 + 0.01%) adult ovaries and chemoablated (0.03 + 0.017%) mouse ovary. The VSELs survive chemotherapy and 48 h of PMSG treatment to chemoablated ovary resulted in increase in their numbers (0.08 + 0.03%) accompanied by initiation of neo-oogenesis in the OSE layer. The process was modulated by FSH both in culture of mechanically isolated OSE cells and adult chemoablated intact ovary culture. Successful formation of germ cell nests was observed and the manuscript was accepted for publication after a very strict review process and only after we inserted images of 4 different germ co-expressing OCT-4, PCNA and DAPI to convince the reviewers.

Readers may wonder why we did not observe complete neo-oogenesis and follicle assembly from the stem cells in chemoablated ovary. The response is simple that chemotherapy affects the ovarian stem cell niche similar to the report in testes [60]. Thus we saw self-renewal, clonal expansion, proliferation, and initial differentiation of stem cells into oocytes but the process of differentiation and PF assembly could not be completed because of the compromised niche. We recently achieved spectacular success of restoring spermatogenesis in chemoablated testis by providing a healthy niche by way of transplanting Sertoli or mesenchymal cells [61]. Transplanted cells act as a source of growth factors and cytokines essential for differentiation of surviving VSELs in chemoablated testes into sperm. Similar experiments could not be attempted in mice ovaries because of their small size which reduces further after chemotherapy. Here we encourage the readers to refer to the beneficial effects of mesenchymal cells (MSCs) transplantation to improve ovarian function reviewed recently [36]. It is intriguing that transplanting MSCs restores normal ovarian function/morphology (but do not themselves form oocytes) and rather endogenous oogenesis is restored (possibly from the VSELs that survive cytotoxic insult). Similarly, Tilly's group reported that transplanting bone marrow or peripheral blood cells in chemoablated ovary resulted in restoration of function and formation of oocytes from endogenous stem cells [18]. All this exciting data emerging from various laboratories suggests that a quiescent population of VSELs in adult mammalian ovary which survive oncotherapy should be appreciated and further exploited to restore ovarian function. VSELs may have a role in giving rise to PCOS was shown by our group [49], but needs to be investigated in details.

Zhang et al. [46] further state that rather than the ovarian stem cells, embryonic and induced pluripotent stem cells (iPS) have a promising future to generate oocytes for fertility treatment. Research efforts to make oocytes using mES cells are ongoing for last 30 years and recent efforts for using hES cells are ongoing for more than 15 years. Similarly iPS cells have also been used to make gametes* in vitro* but as concluded recently obtaining gametes from these stem cells remains a distant dream [62]. We recently discussed and compared the ovarian/testicular VSELs with ES/iPS cells and why VSELs (isolated from adult human, sheep, monkey, and rabbit OSE) and from chemoablated mice ovaries spontaneously differentiate into oocytes [16, 63]. Also for the first time, we succeeded to spontaneously differentiate testicular VSELs enriched from chemoablated mouse testis into sperm [64]. It is difficult to convert ES/iPS cells into PGCs as they have very distinct epigenetic status which cannot be replicated easily in a culture dish. On the other hand, VSELs are considered equivalent to PGCs and show excellent ability to differentiate into gametes.

## 5. Conclusions

To conclude, data emerging from various laboratories suggests the presence of stem cells in adult ovary surface epithelium. Our group has shown that stem cells in the OSE comprise smaller VSELs and slightly larger progenitors (OGCSs/OSCs) equivalent to SSCs in the testes. They have the ability to undergo postnatal oogenesis, differentiate into oocytes, and undergo PF assembly under the influence of FSH which acts through alternatively spliced FSHR3 isoform. This increasing understanding of ovarian stem cell biology led to initial success of restoration of function in chemoablated adult mice ovary. Further studies are warranted to confirm this amazing success achieved by our group and others in the field. A similar FSH-FSHR3-stem cell axis has recently been deciphered in mouse testis as well [65].

## Figures and Tables

**Table 1:** 

Group	Year	Studies conducted	References
Jonathan L. Tilly	2004	They challenged the central dogma of fixed number of follicles. Cells in ovary surface epithelium expressed SCP3, MVH-BrdU.	Johnson et al. [2]
	2005	Bone marrow and peripheral blood were shown as a source of germ cells. Germ line markers were found in the bone marrow. Bone marrow and peripheral blood transplantation restored oocyte production in chemoablated (and also after total body irradiation) wild-type and mutant mice. Bone marrow transplantation in chemoablated ovaries resulted in formation of oocytes, but surprisingly from the endogenous cells.	Johnson et al. [18]
	2007	Aged mouse ovaries harbor stem cells expressing Stra8 and Dazl but no oocytes. These stem cells retain the ability to undergo neo-oogenesis when grafted in young wild type mice.	Lee et al. [19]
	2009	Germ line stem cells were isolated from adult mouse ovary and human cortical tissue by FACS using DDX4 as a marker. These cells could be expanded for months *in vitro* and spontaneously differentiated into 35–50 *µ*m oocytes. Human cells from reproductive age group were tagged with GFP and injected into human cortical tissue and resulted in GFP positive oocytes in immunodeficient mice.	Niikura et al. [20]
	2012	They described and validated FACS based protocol to isolate rare mitotically active germline stem cells from adult mouse ovaries and human ovarian cortical tissue, which upon further passage could give rise to 35–50-*µ*m oocytes *in vitro* validated by various methods, as well as generated oocytes *in vivo* upon xenotransplantation into immunodeficient female mice.	White et al. [21]
	2013	Gene expression profiles of Ddx4 sorted OSCs and cultured OSCs, ESCs, PGCs, and SSCs were compared. OSCs expressed germline markers but distinct signatures as compared to that of PGCs and SSCs. *In vitro* culture of OSCs triggered pluripotency gene expression similar to PGCs	Imudia et al. [22]
	2013	Bone morphogenetic protein 4 promotes mammalian oogonial stem cell differentiation and results in increased expression of meiosis specific markers (Stra8, Msx1, and Msx2) via Smad 1/5/8 activation	Park et al. [23]

Antonin Bukovsky	2004	Mesenchymal cells in tunica albuginea are bipotent progenitors which can differentiate into both granulosa and germ cells. They studied adult human ovarian tissue and concluded that granulosa cells originate in the OSE by epithelial-mesenchymal transition.	Bukovsky et al. [24]
	2005	Cultured adult human OSE cells in medium without phenol red led to development of granulosa-like cells and epithelial and neural and mesenchymal type cells. When OSE cells were cultured in the presence of phenol red medium, it resulted in the formation of >180 *µ*m oocyte-like structures which exhibited germinal vesicle breakdown, polar body, and surface expression of zona pellucida proteins.	Bukovsky et al. [25]
	2008	Described neo-oogenesis and follicular assembly in adult ovary. Observed expression of meiotic entry synaptonemal complex protein 3 (SCP3)—a marker for meiosis in OSE.	Bukovsky et al. [26]
	2012	Follicular renewal in rodents is initiated by bone marrow derived cells related to the immune system, which interact with OSE cells in normal adult rats or medullary sex cord cells in adult neonatally estrogenized rats lacking OSE.	Bukovsky and Caudle [27]

Irma Virant-Klun	2008	OSE cells from 20 postmenopausal and 5 women with premature ovarian failure were used to isolate putative stem cells. Small round cells with a bubble-like structure, 2 to 4 *µ*m in size, were detected which expressed pluripotent markers SSEA4, Oct-4, Nanog, Sox-2, and c-kit.	Virant-Klun et al. [28]
	2009	Ovarian tissue from postmenopausal women was cultured to study oogenesis *in vitro*. Small round cells with a bubble-like structure and with a diameter from 2 to 4 *μ*m were isolated from OSE. They expressed pluripotent markers including SSEA-4, Oct-4, Nanog, Sox-2, and c-kit. These cells on culture proliferated and formed embryoid body-like structures but did not form teratoma in SCID mice. Cells grew in size and reached a diameter of approximately 95 *µ*m and expressed Oct-4, c-kit, VASA, and ZP2. Few expressed a zona pellucida-like structure, germinal vesicle, and polar body-like structures. Parthenote embryos were also observed which expressed Oct-4, Sox-2, and Nanog and were normal for chromosomes X, 13, 16, 18, 21, and 22.	Virant-Klun et al. [29]
	2011	Small, round SOX-2 positive stem cells were detected in the OSE of women with premature ovarian failure and high FSH and LH. On culture with follicular fluid to provide ovarian niche, primitive oocyte-like structures and cell clusters were observed which were alkaline phosphatase positive and expressed pluripotent markers SOX-2 and SSEA-4. Single oocyte-like cells expressed genes *OCT-4A, SOX-2, NANOG, NANOS, STELLA, CD9, LIN28, KLF4, GDF3, *and *MYC*, characteristic for pluripotent stem cells.	Virant-Klun et al. [30]
	2013	Small putative SSEA-4 positive stem cells up to 4 *µ*m in size were isolated by FACS and MACS from cultures of human cortical tissue. They expressed pluripotent markers but were relatively low compared to hES cells. In addition, they expressed genes of primordial germ cell lineages VASA, PRDM1, PRDM14, and DPPA3.	Virant-Klun et al. [31, 32]
	2013	Ovarian cell cultures could be established from cortical biopsies and the cells expressed pluripotent markers (alkaline phosphatase, SSEA-4, OCT-4, SOX-2, NANOG, LIN28, and STELLA), germinal lineage (DDX4/VASA) and multipotency (M-CAM/CD146, Thy-1/CD90, and STRO-1). These cells were SSEA-4 positive, spherical in shape, and small up to 4 *µ*m in size. These cells could be differentiated into 3 germ layers but did not form teratoma in immunodeficient mice.	Stimpfel et al. [33]

Deepa Bhartiya	2011	Two distinct populations of stem cells were detected in OSE isolated from adult rabbit, monkey, sheep, and human ovaries. Spherical cells with high N/C ratio were smaller than RBCs in size and expressed nuclear OCT-4 and SSEA-4 and the bigger cell population expressed cytoplasmic OCT-4 and minimal SSEA-4. Oct-4, Oct-4A, Nanog, Sox-2, TERT, and Stat-3 were detected in human and sheep OSE cells by RT-PCR. The stem cells underwent spontaneous differentiation into oocyte-like structures, parthenote-like structures, embryoid body-like structures, cells with neuronal-like phenotype, and embryonic stem cell-like colonies, whereas the epithelial cells transformed into mesenchymal phenotype by epithelial-mesenchymal transition. Oocyte-like structures expressed c-KIT, DAZL, GDF-9, VASA, and ZP4.	Parte et al. [13]
	2012	PMSG (5IU) was observed to exert direct proliferative effect on OSE and increased expression of FSHR and PCNA. OSE appeared multilayered at several positions and MVH positive germ cell nests and cohort of newly formed PF were visualized along with increased expression of Oct-4A, Nanog, Scp-3, Oct-4B, and Mvh. Study provided first evidence that stem cells activity in OSE and neo-oogenesis is modulated by FSH in adult mammalian ovaries. Similar findings were also observed in normal estrus cycle coinciding with the proestrus peak of FSH and numbers of cohorts along the surface of the ovary were increased after PMSG treatment.	Bhartiya et al. [15]
	2013	Effect of FSH and bFGF was studied on human and marmoset ovarian cortical tissues in organ culture format. Ovarian stem cells were found to be released on the culture inserts and retained the potential to spontaneously differentiate into oocyte-like structures in extended cultures. Both FSH and bFGF induced proliferation of OSE along with increased expression of gene transcripts specific for pluripotent stem cells (Oct-4A and Nanog) suggestive of VSELs and early germ cells (Oct-4, c-Kit, and Vasa) suggestive of OGSCs and follicular transition (oocyte-specific Gdf-9 and Lhx8, and granulosa cell specific Amh).	Parte et al. [17]
	2013	Effect of FSH was studied on sheep stem cells in OSE *in vitro*. FSH increased stem cells self-renewal and clonal expansion evident by the appearance of stem cell clusters. FSH receptors were expressed on ovarian stem cells whereas the epithelial cells were distinctly negative. An increase in R3 mRNA transcripts was noted after 3 hrs of FSH treatment and was reduced to basal levels by 15 hrs, whereas R1 transcript expression remained unaffected. FSHR and OCT4 were immunolocalized in nuclei of stem cells and showed nuclear or ooplasmic localization in oocytes of primordial follicles and in cytoplasm of granulosa cells in growing follicles. Thus FSH appears to modulate ovarian stem cells activity via FSH-R3 to undergo potential self-renewal, clonal expansion as “cysts,” and differentiation into oocytes. OCT-4 and FSHR proteins (required initially to maintain pluripotent state of VSELs and for FSH action, respectively) gradually shift from nuclei to cytoplasm of developing oocytes and are later possibly removed by surrounding granulosa cells as the oocyte prepares itself for fertilization.	Patel et al. [14]
	2013	A genetic lineage tracing study which failed to detect stem cells and germ cell clusters in adult mouse ovary was challenged. Cysts were observed and confocal microscopy imaging confirmed cytoplasmic continuity amongst the cells comprising the cysts. Germ cell nests expressed PCNA and SSEA-4 suggestive of their germ cell characteristic and rapid mitotic division.	Bhartiya et al. [34]
	2014	Ovarian stem cells and germ cell clusters were enriched by immunomagnetic sorting using SSEA-4 and were further characterized. Differential expression of markers specific for pluripotent VSELs (nuclear OCT-4A, SSEA-4, and CD133), OGSCs (cytoplasmic OCT-4) primordial germ cells (FRAGILIS, STELLA, and VASA), and germ cells (DAZL, GDF-9, and SCP-3) were studied. Within one week of culture, stem cells became bigger in size, developed abundant cytoplasm, differentiated into germ cells, revealed presence of Balbiani body-like structure (mitochondrial cloud), and exhibited characteristic cytoplasmic streaming.	Parte et al. [35]
	2015	Study was undertaken to investigate stem cells in adult mouse ovary, the effect of chemotherapy on them, and their potential to differentiate into germ cells. VSELs in adult mouse ovary were SCA-1+/Lin−/CD45− and positive for nuclear OCT-4, Nanog, and SSEA-1. VSELs survived chemotherapy and OSE culture of chemoablated ovary OSE resulted in appearance of proliferating germ cell clusters and MVH and GDF9 positive oocyte-like structures spontaneously differentiated by day 6. FSH exerted a direct stimulatory action on the OSE and induced stem cell proliferation and differentiation into premeiotic germ cell clusters during intact chemoablated ovary culture. PMSG treatment to chemoablated mice resulted in self-renewal of VSELs (LIN−/CD45−/SCA1+) that were 0.02 ± 0.008% in normal ovary and 0.03 ± 0.017% in chemoablated ovary and PMSG treatment to chemoablated ovary increased VSELs to 0.08 ± 0.03%.	Sriraman et al. [16]

## Abbreviations

ES cells: Embryonic stem cells
FSH: Follicle stimulating hormone
FSHR: FSH receptor
FSHR1: Canonical FSHR, acts via cAMP pathway
FSHR3: Alternately spliced FSHR transcript, acts via MAPK pathway
iPS: Induced pluripotent stem cells
MSCs: Mesenchymal stem cells
OGSCs: Ovarian germ stem cells
OSCs: Oogonial stem cells
OSE: Ovary surface epithelium
PCOS: Polycystic ovarian syndrome
PF: Primordial follicles
PGCs: Primordial germ cells
POF: Premature ovarian failure
SSCs: Spermatogonial stem cells
VSELs: Very small embryonic-like stem cells.
